# Diagnostic efficacy of cytologic smear and pathologic histology in the differential diagnosis of distal biliary stricture via EUS-guided fine-needle aspiration

**DOI:** 10.1097/eus.0000000000000093

**Published:** 2025-12-13

**Authors:** Zheng Liang, Peng Li, Xiao Han, Shutian Zhang, Yongqiu Wei

**Affiliations:** 1 Department of Gastroenterology, Beijing Friendship Hospital, Capital Medical University, National Clinical Research Center for Digestive Disease, Beijing, China; 2 Department of Hepatology, Beijing Friendship Hospital, Capital Medical University, National Clinical Research Center for Digestive Disease, Beijing, China.

**Keywords:** Distal biliary stricture (DBS), EUS-guided fine-needle aspiration (EUS-FNA), Differential diagnosis, Histopathology, IgG4-related disease (IgG4-RD)

## Abstract

**Background and Objectives:**

Distal biliary strictures (DBSs) can be caused by various malignancies, making accurate and early diagnosis crucial. Histopathology is the gold standard for diagnosis, with several methods available for tissue sampling. This study evaluates the performance of EUS-guided fine-needle aspiration (EUS-FNA) cytologic smears and histopathology in diagnosing suspected malignant DBSs.

**Methods:**

A retrospective cohort study was conducted on patients who underwent EUS-FNA between January 2017 and January 2023 for DBSs. Demographic, imaging, procedural, and clinical data were collected. The diagnostic performance of EUS-FNA cytology, histology, and their combination was assessed in terms of sensitivity, specificity, positive predictive value, and negative predictive value. Subgroup analyses were conducted based on imaging and endoscopy characteristics.

**Results:**

EUS-FNA for cytology had a sensitivity of 69.1% and specificity of 97.5%. EUS-FNA histology had a sensitivity of 76.4% and specificity of 99.1%. There was no difference in diagnostic efficacy between the two above (*P* > 0.05). Combining cytology and histology improved sensitivity to 82%. When 20 cases (6.8%) with histological slide failures were considered as negative, histologic sensitivity was 69.1%, completely consistent with cytology alone (*P* = 1). The presence of a mass shadow on computed tomography or magnetic resonance imaging was associated with higher cytologic diagnostic sensitivity compared with simple stenosis without a mass shadow (57.4% *vs.* 75.9%, *P* = 0.011). The larger the mass, the higher the cytologic diagnostic sensitivity. The radiologist’s diagnostic imaging tendencies, that is, malignant, benign, and indeterminate, also affected cytologic diagnostic sensitivity (78.2% *vs.* 63.9% *vs.* 51.9%, *P* = 0.002). Furthermore, among our cohort of 118 patients diagnosed with benign DBSs, a notable subset of 33 individuals (28%) received a diagnosis of IgG4-related disease.

**Conclusion:**

EUS-FNA histology combined with cytology was a reliable diagnostic method. There is no difference in diagnostic efficacy between EUS-FNA cytology and histology, irrespective of considering instances of histological slide failure. The presence of a mass shadow on computed tomography or magnetic resonance imaging and the size of the mass influenced the diagnostic efficacy of cytology. Additionally, IgG4-related diseases, accounting for a significant proportion of cases, were important in the differential diagnosis of these strictures.

## INTRODUCTION

The extrahepatic bile duct consists of perihilar and distal segments, with the division occurring near the duodenal junction where the cystic duct joins the common bile duct.^[1]^ Pancreatic ductal adenocarcinoma, cholangiocarcinoma (CCA), and ampullary cancer, collectively part of a cancer superfamily, are common causes of malignant distal biliary strictures (DBSs).^[2]^ Surgical resection is feasible in only a small percentage (10%–15% for pancreatic ductal adenocarcinoma, 20%–49% for CCA), often leaving advanced stages for palliative care,^[3–5]^ and a significant percentage (15%–24%) of benign bile duct stricture cases undergo unnecessary surgery due to misdiagnosis, causing substantial physical, psychological, and financial burdens.^[6]^ Hence, early and accurate differential diagnosis for DBSs is vital.

Histopathology stands as the definitive diagnostic modality.^[7]^ At present, 3 foremost approaches are accessible for the pathological assessment of suspected malignant biliary strictures: tissue sampling via endoscopic retrograde cholangiopancreatography (ERCP), peroral or percutaneous transhepatic cholangioscopy (POCS or PTCS), and EUS-guided fine-needle aspiration (EUS-FNA) or biopsy.^[8]^

ERCP and POCS or PTCS-based tissue sampling techniques allow for the acquisition of histological specimens from stenotic sites, guided by fluoroscopy and direct visualization, respectively.^[8]^ These methods exhibit sensitivities ranging from 30% to 78% for ERCP and from 80% to 100% for POCS or PTCS.^[9–14]^ Nevertheless, when addressing DBSs primarily attributed to pancreatic cancer, these aforementioned techniques present certain limitations, especially in cases where the tumor has not infiltrated the bile duct. A recent study underscored this challenge, revealing an accuracy rate of 82.4% for intraductal lesions in contrast to 54.8% for extraductal lesions.^[15]^

EUS-FNA offers the advantage of visualizing the bile duct stricture and adjacent organs for targeted tissue acquisition.^[16,17]^ Sensitivity for diagnosing malignant biliary stricture varies (27%–83%), with lower sensitivity for distal (59%) compared with proximal (81%) stricture.^[18,19]^ Operator experience and lesion nature can lead to inadequate tissue samples. In such cases, cytologic smears obtained via EUS-FNA serve as a complementary tool during the same procedure.^[20,21]^ This study assesses the performance of EUS-FNA cytologic smears and histopathology in concurrent procedures, focusing on their combined diagnostic utility for suspected malignant DBSs.

## METHODS

### Study design and patients

This retrospective cohort study received ethical approval from the Institutional Review Board of Beijing Friendship Hospital, Capital Medical University (protocol no. MR-11-23-016475), and adhered to the principles outlined in the 1975 Declaration of Helsinki (revised 2013, version 7). The “Medcare Smart Gastroenterology Specialized Diagnosis and Treatment Platform” database was used to identify adult patients who underwent EUS-FNA from January 2017 until January 2023 at the Gastrointestinal Endoscopy Center, Beijing Friendship Hospital.

Inclusion criteria for this investigation encompassed the following: (1) patients with a confirmed diagnosis of DBSs through imaging modalities such as abdominal ultrasound, computed tomography (CT), and MRCP; (2) patients who provided informed consent for EUS-FNA and subsequently underwent the procedure; (3) patients subjected to concurrent cytologic smears and histopathologic preparations during the EUS-FNA process; and (4) patients with a follow-up duration exceeding 12 months. Exclusion criteria comprised (1) instances of EUS-FNA failure and (2) cases with indeterminate final diagnoses. For eligible patients meeting the inclusion criteria, we accessed electronic medical records to gather demographic data, imaging findings, procedural specifics, cytology and histology results, as well as clinical diagnoses pertinent to DBSs.

### Endoscopic procedures

Endoscopic procedures adhered to standardized protocols under the guidance of 10 seasoned endoscopists, each boasting extensive experience, with an annual caseload of 400 EUS procedures. Prior to the commencement of any procedure, written informed consent was meticulously obtained from each patient. EUS-FNA was conducted using the Olympus GF-UCT260 linear-array echoendoscope (Olympus, Tokyo, Japan) and the Pentax EG-3870UTK echoendoscope (Pentax, Tokyo, Japan). Prior to FNA, Doppler examination was diligently performed to ascertain the absence of intervening vascular structures along the intended needle trajectory.

EUS-FNA was carried out using either a standard 22- or 25-gauge needle (Echotip; Cook Medical, Bloomington, IN), as determined by the endoscopist, considering lesion characteristics and location. Following each lesion puncture, meticulous strokes ranging from 10 to 30 were meticulously administered. Rapid on-site evaluation (ROSE) was not consistently available as a routine practice. Puncture procedures were iteratively performed until the appearance of macroscopically visible whitish material, typically involving 1 to 4 passes. FNA samples were meticulously transferred onto glass slides and subsequently fixed with 95% ethanol for subsequent Papanicolaou staining. Any biliary biopsies and visible core specimens were promptly placed in formalin for subsequent microhistological analysis. All samples were promptly dispatched to the pathology department for meticulous interpretation by expert pathologists and cytopathologists with dedicated expertise in biliopancreatic diseases (see the Supplemental material for details, http://links.lww.com/ENUS/A363).

### Study definitions

Pathological diagnostic results obtained through cytology and histology were categorized into 4 distinct groups: “negative,” “nature to be determined,” “suspected malignancy,” and “malignant.” For the purpose of our analysis, we combined “suspected malignancy” and “malignant” categories into a single entity termed “malignant tumors.” The definitive clinical diagnosis was established based on histopathologic findings and was corroborated with clinical or imaging follow-up, a methodology consistent with previous studies.^[22]^ We characterized DBSs as “malignant” if they met any of the following criteria: the diagnosis of adenocarcinoma was confirmed via histological specimens (including ERCP-based transpapillary biopsy sampling, EUS-FNA histopathology, or surgical specimens), or if clinical or radiographic malignancy progression after ≤12 months of follow-up, or if patients succumbed to bile duct malignancy at ≤12-month follow-up from the initial consultation.^[22]^ Conversely, benign DBSs were defined under the following circumstances: no discernible clinical or imaging progression longer than a 12-month follow-up, resolution of the stricture site within the same timeframe, or surgical resection specimens that confirmed the presence of a benign bile duct lesion. Furthermore, diagnostic criteria according to the 2019 American College of Rheumatology/European League Against Rheumatism (ACR/EULAR) classification criteria for IgG4-Related Disease for DBSs due to IgG4-related diseases (IgG4-RDs) were used.^[23]^

### Statistical analysis

Baseline characteristics were summarized as follows: continuous variables were presented as mean ± standard deviation or as median with interquartile range, whereas categorical variables were reported as counts and percentages. The diagnostic performance of each biliary test was evaluated in terms of sensitivity, specificity, positive predictive value (PPV), and negative predictive value (NPV). These metrics were calculated with respect to the final clinical diagnosis in both cohorts, utilizing the exact binomial test, and were reported along with 95% confidence intervals (CIs). To assess the comparability of sensitivities between different tests, the exact McNemar test was used. Statistical significance was determined based on 2-sided *P* values, with a threshold of *P* < 0.05 considered as indicative of statistical significance. All statistical analyses were conducted using the R statistical software (http://www.R-project.org) and Statistical Package for Social Sciences (version 25.0; SPSS Inc, Chicago, IL).

## RESULTS

### Clinicopathological characteristics of the included patients

**Figure 1:**
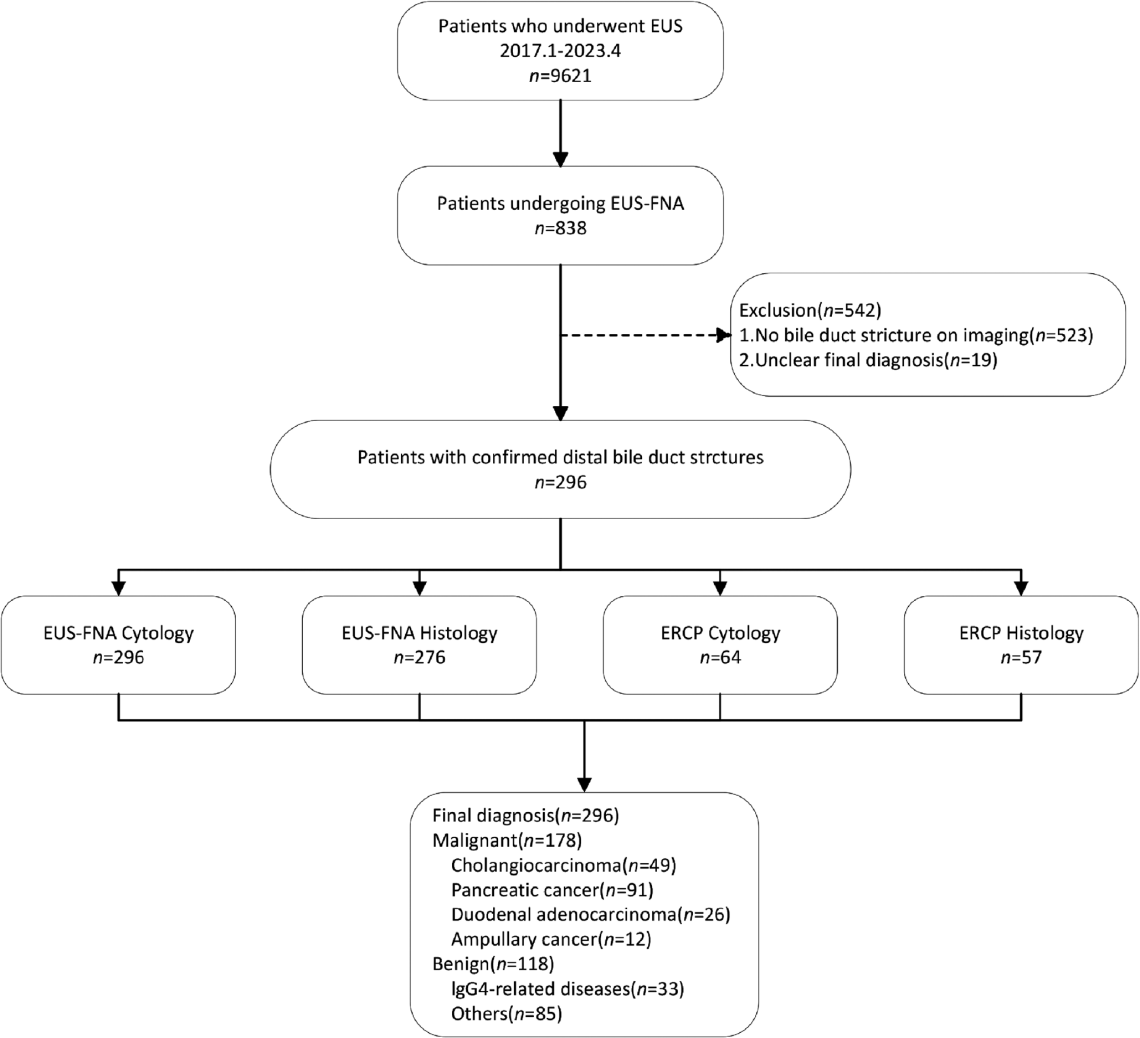
Flow diagram for 296 patients referred for EUS-FNA. EUS-FNA: EUS-guided fine-needle aspiration.

A total of 296 patients presenting with DBSs between January 2017 and January 2023 were included in this study. All patients underwent successful EUS-FNA smear cytology procedures. However, it is noteworthy that 20 patients failed to obtain sufficient histologic specimens for analysis. Additionally, 64 patients underwent concurrent ERCP brushing, whereas 57 patients underwent ERCP biopsy. Based on established diagnostic criteria, our cohort comprised 178 patients diagnosed with malignant DBSs, further categorized as follows: 91 cases of pancreatic carcinomas, 49 of CCAs, 26 of duodenal adenocarcinomas, and 12 of ampullary cancer. During follow-up, 118 patients received a diagnosis of benign DBSs, with 33 cases attributed to IgG4-RD [Figure 1].

Table 1 provides an overview of essential patient information, complication, and their tumor histories, categorized by benign and malignant DBSs. Meanwhile, Table 1 also presents a comprehensive array of laboratory indicators and imaging characteristics.

**Table 1:** Baseline characteristics of the included patients

Characteristics	Malignancy (*n* = 178)	Benign distal bile duct stricture			P*	*P* ^†^	*P* ^‡^	*P* ^§^
		IgG4-related disease (*n* = 33)	Others (*n* = 85)	All benign (*n* = 118)				
Male sex (male %)	113 (63.5)	26 (78.8)	55 (64.7)	81 (68.6)	0.384	0.102	0.11	0.891
Age (y)	64.78 ± 10.71	63.12 ± 9.25	61.42 ± 12.11	61.9 ± 11.37	0.572	0.102	0.289	0.237
BMI (kg/m^2^)	23.61 ± 4.25	22.65 ± 2.65	23.27 ± 2.51	23.08 ± 3.27	0.208	0.224	0.404	0.471
Complication								
Acute cholangitis	35 (19.7)	5 (15.2)	23 (27.1)	28 (23.7)	0.403	0.129	0.636	0.204
Acute pancreatitis	16 (9.0)	10 (30.3)	23 (27.1)	33 (28.0)	<0.001	0.82	0.001	<0.001
Bile duct stones	17 (9.6)	1 (3.0)	31 (36.5)	32 (27.1)	<0.001	<0.001	0.218	<0.001
Past Medical History								
Personal history of malignant tumors	15 (8.4)	-	6 (7.1)	6 (5.1)	0.062	0.184	0.084	0.811
Family history of malignant tumors^∥^	23 (12.9)	3 (9.1)	5 (5.9)	8 (6.8)	0.019	0.684	0.539	0.091
Hypertension	77 (43.3)	13 (39.4)	40 (47.1)	53 (44.9)	0.779	0.538	0.68	0.597
Diabetes	50 (28.1)	10 (30.3)	14 (16.5)	24 (20.3)	0.132	0.126	0.796	0.046
Drinking	40 (22.5)	14 (42.4)	28 (32.9)	42 (35.6)	0.014	0.393	0.016	0.073
Smoking	67 (37.6)	19 (57.6)	38 (44.7)	57 (48.3)	0.069	0.225	0.036	0.284
Laboratory test results								
ALT (U/L)	100.00(38.75,224.00)	131.00(55.50,250.00)	28.00(16.00,93.5)	47.50(16.75,154.00)	<0.001	<0.001	0.200	<0.001
AST (U/L)	83.40(36.98,151.23)	98.35(53.68,225.00)	26.10(18.90,50.40)	34.00(20.00,86.75)	<0.001	<0.001	0.118	<0.001
ALP (U/L)	362.00(194.00,535.00)	447.00(238.00,659.00)	120.00(81.00,190.00)	142.00(91.00,335.00)	<0.001	<0.001	0.328	<0.001
GGT (U/L)	441.00(175.50,883.00)	452.00(298.75,930.00)	116.00(25.00,457.50)	206.00(29.00,579.00)	<0.001	<0.001	0.589	<0.001
Tbil (μmol/L)	122.30(22.61,254.47)	107.72(39.40,176.77)	18.00(12.79,43.22)	26.39(15.42,84.94)	<0.001	<0.001	0.576	<0.001
Dbil (μmol/L)	70.98(8.02,136.12)	53.81(18.85,113.19)	5.80(2.70,18.99)	9.10(3.24,45.25)	<0.001	<0.001	0.630	<0.001
Ibil (μmol/L)	54.20(15.18,106.03)	36.11(20.16,70.15)	13.99(9.23,24.51)	16.95(10.99,36.17)	<0.001	<0.001	0.355	<0.001
CRP (mg/L)	7.99(2.40,16.56)	5.40(1.04,8.72)	3.53(1.00,17.50)	4.00(1.00,10.99)	0.001	0.954	0.020	0.008
IgG4 (g/L)	0.37(0.24,0.58)	6.06(3.36,10.11)	0.68(0.40,1.06)	2.28(0.68,7.24)	<0.001	<0.001	<0.001	0.002
CA199 (U/mL)	207.80(51.75,946.57)	69.30(20.99,137.70)	19.50(7.95,59.80)	27.75(9.15,92.03)	<0.001	0.002	0.001	<0.001
CEA (ng/mL)	2.87(1.83,5.28)	2.32(1.52,3.57)	1.82(1.27,2.89)	1.85(1.32,3.03)	<0.001	0.099	0.002	<0.001
CA125 (U/mL)	17.20(11.08,33.68)	15.00(10.10,24.72)	9.60(6.41,15.15)	11.20(6.98,19.41)	<0.001	0.002	0.318	<0.001
CA153 (U/mL)	6.75(5.10,11.85)	5.70(4.10,8.10)	6.70(4.90,9.90)	6.30(4.53,9.28)	0.167	0.302	0.122	0.366
CA724 (U/mL)	2.53(1.52,4.33)	1.43(0.93,2.05)	2.06(1.35,3.10)	1.79(1.10,2.91)	0.001	0.008	<0.001	0.054
CA50 (U/mL)	95.49(27.65,359.24)	30.13(9.42,117.65)	10.83(4.46,34.07)	12.97(5.63,44.37)	<0.001	0.002	0.009	<0.001
CA242 (U/mL)	28.65(4.56,195.03)	4.07(2.49,5.10)	4.27(2.42,9.03)	4.15(2.59,7,89)	<0.001	0.467	0.002	<0.001
Radiology								
Expansion diameter^¶^	1.64 ± 0.49	1.42 ± 0.39	1.45 ± 0.40	1.44 ± 0.40	0.146	0.928	0.348	0.207
MRCP traction^¶^	43 (24.2)	5 (15.2)	13 (15.3)	10 (8.5)	<0.001	0.111	0.227	<0.001
Area^¶^	7.78(4.50,13.73)	9.92(2.42,13.60)	4.15(1.80,11.18)	4.65(2.10,12.69)	0.051	0.395	0.647	0.037
Radiographic report^¶^					<0.001	<0.001	<0.001	<0.001
Malignant	111 (62.4)	4 (12.1)	4 (4.7)	8 (6.8)				
Benign	11 (6.2)	24 (72.7)	34 (40.0)	58 (49.2)				
Uncertainty	54 (30.3)	5 (15.2)	47 (55.3)	50 (42.4)				

*P: all benign patients *versus* all malignant patients.

^†^*P*: IgG4-related disease *versus* other benign patients.

^‡^*P*: IgG4-related disease *versus* all malignant patients.

^§^*P*: other benign patients *versus* all malignant patients.

^∥^Family history of malignant tumors: immediate family member (parent, sibling or child) with a malignant tumor.

^¶^Expansion diameter: maximum dilated diameter of the bile duct above the site of stenosis; MRCP traction: MRCP image showing bile duct amputation; area: cross-sectional area of mass-type lesions calculated on CT or MRI images; radiographic report: diagnostic tendency after review of films (CT or MRI) by 2 or more specialized radiologists. BMI: body mass index; ALT: alanine aminotransferase; AST: aspartate aminotransferase; ALP: alkaline phosphatase; GGT: gamma-glutamyl transferase; Tbil: total bilirubin; Dbil: direct bilirubin; Ibil: indirect bilirubin; CRP: c-reactive protein; IgG4: Immunoglobulin g4; CA199: carbohydrate antigen 199; CEA:carcinoembryonic antigen; CA125: carbohydrate antigen 125; CA153: carbohydrate antigen 153; CA724: carbohydrate antigen 724; CA50: carbohydrate antigen 50; CA242: carbohydrate antigen 242.

### Key findings: diagnostic effectiveness

The diagnostic performance of EUS-FNA cytology yielded noteworthy results with a sensitivity of 69.1% (95% CI, 61.7%–75.7%) and specificity of 97.5% (95% CI, 92.2%–99.3%). In contrast, EUS-FNA histology demonstrated a sensitivity of 76.4% (95% CI, 68.9%–82.6%) and specificity of 99.1% (95% CI, 94.5%–100%). Although there was a 7.3% improvement in sensitivity when comparing cytologic with histologic results, this difference did not reach statistical significance (*P* = 0.133). However, when combining the cytology and histology results of EUS-FNA, diagnostic sensitivity and specificity improved to 82% (95% CI, 75%–87.4%), 99.1% (95% CI, 94.5%–100%), respectively. The combined approach’s diagnostic sensitivity was significantly higher compared with cytology results alone (*P* = 0.006) but showed no statistically significant difference compared with histology results (*P* = 0.216) [Table 2].

**Table 2:** Performance characteristics of tissue sampling techniques for malignancy detection

Diagnostic approaches	Sensitivity (%)	Specificity (%)	Positive predictive value (%)	Negative predictive value (%)
EUS-FNA cytology (*n* = 296)	69.1 (61.7–75.7)	97.5 (92.2–99.3)	97.6 (92.7–99.4)	67.6 (60.0–74.5)
EUS-FNA histology* (*n* = 276)	76.4 (68.9–82.6)^†^	99.1 (94.5–1)^‡^	99.2 (94.9–1)	75.0 (67.2–81.5)
EUS-FNA cytology and histology* (*n* = 276)	82.0 (75.0–87.4)^§,∥^	99.1 (94.5–1)	99.2 (95.3–1)	79.7 (72.0–85.8)
EUS-FNA histology^¶^ (*n* = 296)	69.1 (61.7–75.7)^#,**^	99.2 (94.7–1)	99.2 (94.9–1)	68.0 (60.4–74.8)
EUS-FNA cytology and histology^¶^ (*n* = 296)	73.6 (66.4–79.8)^f,g,h^	98.3 (93.4–99.7)	98.4 (94.1–99.7)	71.2 (63.5–77.8)
BC (*n* = 64)	50.0 (34.8–65.2)	95.0 (73.1–1)	95.7 (76.0–99.8)	46.3 (31.0–62.4)
TPB (*n* = 57)	70.0 (50.4–84.6)	96.3 (79.1–99.8)	95.5 (75.1–99.8)	74.3 (56.4–86.9)
BC + TPB (*n* = 32)	77.8 (51.9–92.6)	1 (73.2–1)	1 (73.2–1)	77.8 (51.9–92.6)
EUS-FNA + BC + TPB (*n* = 30)	88.2 (62.3–97.9)	92.3 (62.1–99.6)	93.8 (67.7–99.7)	85.7 (56.2–97.5)

*Patients with slide failures, resulting in insufficient histologic specimens, were excluded.

^†^*P*: EUS-FNA cytology *versus* histology (*) = 0.133.

^‡^*P*: EUS-FNA cytology *versus* histology (*) = 0.328.

^§^*P*: EUS-FNA cytology *versus* cytology and histology (*) = 0.006.

^∥^*P*: EUS-FNA histology (*) *versus* cytology and histology (*) = 0.216.

^¶^Patients with slide failures, resulting in insufficient histologic specimens, were included and were recognized as a negative histology result.

^#^*P*: EUS-FNA cytology *versus* histology (¶) = 1.

**P: EUS-FNA histology (*) *versus* histology (¶) = 0.133.

^††^*P*: EUS-FNA cytology *versus* cytology and histology (¶) = 0.348.

^‡‡^*P*: EUS-FNA histology (*) *versus* cytology and histology (¶) = 0.554.

^§§^*P*: EUS-FNA cytology and histology (*) *versus* cytology and histology (¶) = 0.064. BC: brush cytology; TPB: transpapillary biopsy; EUS-FNA: endoscopic ultrasound-guided fine needle aspiration.

Notably, 20 patients (6.8%) experienced slide failures, resulting in insufficient histologic specimens. When considering these cases as negative, the sensitivity and specificity for histological diagnosis were as follows: 69.1% (95% CI, 61.7%-74.6%) and 99.2% (95% CI, 92.2%-99.3%), respectively. It is worth mentioning that the diagnostic sensitivity of histology was complete agreement with cytology (*P* = 1). Furthermore, when combining cytology and histology, the diagnostic sensitivity and specificity were 73.6% (95% CI, 66.4%–79.8%) and 98.3% (95% CI, 93.4%–99.7%), respectively. No statistically significant differences in diagnostic sensitivity were observed when compared with either cytology alone (*P* = 0.348) or histology (*P* = 0.554) at this stage [Table 3].

**Table 3:** Comparison of sensitivity and specificity of lesions at different sites

		Bile duct* (*n* = 147)	Pancreas* (*n* = 101)	Duodenal papilla* (*n* = 44)	*P*
EUS-FNA cytology	Sensitivity	69.4 (57.3–79.5)	70.5 (59.0–80.0)	69.2 (48.1–84.9)	0.985
	Specificity	98.7 (91.8–1)	1 (82.1–1)	94.4 (70.6–99.7)	0.325
EUS-FNA pathology	Sensitivity	75.4 (62.9–84.9)	74.6 (62.7–83.9)	79.2 (57.3–92.1)	0.904
	Specificity	98.6 (91.7–1)	1 (81.5–1)	94.4 (70.6–99.7)	0.328
EUS-FNA cytology combined with pathology	Sensitivity	80.0 (67.9–88.5)	83.1 (71.9–90.6)	83.3 (61.8–94.5)	0.878
	Specificity	98.6 (91.7–1)	1 (81.5–1)	94.4 (70.6–99.7)	0.327

*Location of etiologic factors leading to distal bile duct stricture.

In addition, we evaluated the diagnostic performance of various combinations of diagnostic tests [Table 2]. For the 64 patients who underwent concurrent ERCP-based brush cytology, the results showed a diagnostic sensitivity of 50% (95% CI, 34.8%–65.2%) and specificity of 95% (95% CI, 73.1%–100%). Similarly, among the 57 patients who underwent concurrent ERCP-based transpapillary biopsy sampling, the diagnostic sensitivity was 70% (95% CI, 50.4%–84.6%), and specificity was 96.3% (95% CI, 79.1%–99.8%). Notably, in 32 patients who underwent both brush cytology and transpapillary biopsy simultaneously, diagnostic performance significantly improved, yielding a sensitivity of 77.8% (95% CI, 51.9%–92.6%) and specificity of 100% (95% CI, 73.2%–100%). Further enhancement of diagnostic accuracy was observed in 30 patients who underwent simultaneous collection of all 4 pathologic specimens, with diagnostic sensitivity reaching 88.2% (95% CI, 62.3%–97.9%) and specificity at 92.3% (95% CI, 62.1%–99.6%).

### Subgroup analysis

The analysis was meticulously stratified based on the primary pathogenetic cause of DBSs [Table 3]. Notably, when we considered the anatomical location of the primary lesions, we found that the sensitivity of EUS-FNA cytology for lesions in the bile ducts, pancreas, and duodenal papilla was 69.4% (95% CI, 57.3%–79.5%), 70.5% (95% CI, 59%–80%), and 69.2% (95% CI, 48.1%–84.9%), respectively. The corresponding specificity values were 98.7% (95% CI, 91.8%–100%), 100% (95% CI, 82.1%–100%), and 94.4% (95% CI, 70.6%–99.7%). Meanwhile, the diagnostic sensitivity of EUS-FNA histology for detecting lesions at these same sites was as follows: 75.4% (95% CI, 62.9%–84.9%) for bile ducts, 74.6% (95% CI, 62.7%–83.9%) for the pancreas, and 79.2% (95% CI, 57.3%–92.1%) for the duodenal papilla. Specificity values remained consistently high at 98.6% (95% CI, 91.7%–100%), 100% (95% CI, 81.5%–100%), and 94.4% (95% CI, 70.6%–99.7%). Furthermore, when combining EUS-FNA cytology and EUS-FNA histology, we observed improved diagnostic sensitivity for lesions at these anatomical sites, with values of 80% (95% CI, 67.9%–88.5%), 83.1% (95% CI, 71.9%–90.6%), and 83.3% (95% CI, 61.8%–94.5%), respectively. Importantly, specificity values remained consistently high at 98.6% (95% CI, 91.7%–100%), 100% (95% CI, 81.5%–100%), and 94.4% (95% CI, 70.6%–99.7%). Although diagnostic sensitivity appeared higher for lesions with primary disease located around the pancreas and duodenum compared with those in the bile ducts, these differences did not attain statistical significance.

Subgroup analyses were conducted based on pertinent imaging manifestations observed in CT or magnetic resonance imaging scans for cases of DBSs [Table 4]. These analyses considered factors such as the presence of a mass, maximum diameter of upstream bile duct dilatation, presence of truncation, and the size of the mass area. Notably, the diagnostic sensitivity of EUS-FNA cytology exhibited significant variation based on the presence of a mass shadow. Specifically, the sensitivity was notably higher in cases with a discernible mass shadow compared with those without (75.9% *vs.* 57.4%, *P* = 0.011). Furthermore, when combining cytology with histology, the diagnostic sensitivity remained significantly higher in the presence of a mass shadow (85.8% *vs.* 72.7%, *P* = 0.043). However, there was no such difference in the sensitivity of histology alone (79.2% *vs.* 67.3%, *P* = 0.095). Notably, the size of the mass also exerted an influence on the diagnostic sensitivity of cytology. Specifically, when the mass area was greater than or equal to 9.5 cm^2^, the diagnostic sensitivity was significantly higher compared with cases with a mass area less than 9.5 cm^2^ (85.4% *vs.* 66.7%, *P* = 0.046). Additionally, the diagnostic tendencies of specialized imaging physicians significantly impacted the sensitivity of cytology diagnosis. Cytology exhibited a diagnostic sensitivity of only 51.9% (95% CI, 38%–65.5%) for lesions that were not definitively diagnosed by imaging. In cases where the imaging diagnosis leaned toward a benign interpretation (63.6%) or a malignant tendency (78.2%), statistically significant differences were observed (*P* = 0.002).

**Table 4:** Subgroup analysis of diagnostic sensitivity based on radiology and EUS-FNA data

Subgroup	Sensitivity*	P*	Sensitivity^†^	*P* ^†^	Sensitivity^‡^	*P* ^‡^
Mass	75.9 (66.9–83.1)	0.011	79.2 (70.1–86.3)	0.095	85.8 (77.4–91.6)	0.043
No mass	57.4 (44.1–69.7)		67.3 (53.2–79.0)		72.7 (58.8–83.5)	
Expansion diameter < 1.56 cm	72.2 (60.2–81.8)	0.532	80.8 (69.2–89.0)	0.111	85.3 (74.2–92.3)	0.256
Expansion diameter ≥ 1.56 cm	67.7 (57.1–76.9)		67.7 (58.9–75.5)		78.0 (67.3–86.1)	
Traction	73.8 (57.7–85.6)	0.454	73.0 (55.6–85.6)	0.74	75.7 (58.4–87.6)	0.306
No traction	67.7 (58.8–75.5)		75.6 (66.7–82.8)		83.2 (75.0–89.2)	
Cross-sectional area <9.5 cm^2^	66.7 (50.4–80.0)	0.046	82.5 (66.7–92.1)	0.462	85.0 (69.5–93.8)	0.484
Cross-sectional area ≥9.5 cm^2^	85.4 (70.1–93.9)		75.7 (58.4–87.6)		91.9 (77.0–97.9)	
Image report malignant	78.2 (69.1–85.3)	0.002	78.0 (68.4–85.4)	0.345	85.0 (76.1–91.1)	0.105
Image report benign	63.6 (31.6–87.6)		80.0 (44.2–96.5)		90.0 (54.1–99.5)	
Image report indeterminate	51.9 (38.0–65.5)		67.3 (52.3–79.6)		71.4 (56.5–83.0)	
Cholangitis	61.8 (43.6–77.3)	0.275	71.9 (53.0–85.6)	0.651	78.1 (59.6–90.1)	0.615
No cholangitis	71.3 (63.1–78.4)		76.0 (67.5–82.9)		82.2 (74.2–88.1)	
Pancreatitis	71.4 (63.7–78.1)	0.091	75.5 (67.6–82.1)	0.75	82.3 (75.0–87.9)	0.471
No pancreatitis	50.0 (25.5–74.5)		71.4 (42.0–90.4)		71.4 (42.0–90.4)	
Bile duct stones	70.0 (62.2–76.8)	0.782	75.3 (67.4–81.9)	0.539	81.5 (74.1–87.3)	0.557
No bile duct stones	64.7 (38.6–84.7)		73.3 (44.8–91.1)		80.0 (51.4–94.7)	
Operating time <37.36 min	67.6 (55.6–77.8)	0.522	74.6 (62.3–84.1)	1	80.6 (68.8–88.9)	0.791
Operating time ≥37.36 min	72.4 (60.7–81.7)		75.0 (62.8–84.4)		82.4 (70.8–90.2)	
Senior endoscopist	68.9 (60.3–76.4)	0.752	74.8 (66.0–82.0)	0.841	80.5 (72.2–86.9)	0.603
General endoscopist	71.4 (55.2–83.8)		76.3 (59.4–88.0)		84.2 (68.1–93.4)	
Olympus	65.8 (56.4–74,2)	0.175	65.0 (55.5–73.4)	0.322	69.2 (79.9–77.3)	0.097
Pentax	75.9 (62.5–85.7)		72.4 (58.9–83.0)		81.0 (68.2–89.7)	
Needle thickness 22-gauge	66.4 (56.5–75.0)	0.335	75.5 (65.6–83.4)	0.74	82.7 (73.4–89.3)	0.572
Needle thickness 25-gauge	73.7 (60.1–84.1)		73.1 (58.7–84.0)		78.8 (64.9–88.5)	

*EUS-FNA cytology.

^†^EUS-FNA pathology.

^‡^EUS-FNA cytology combined with pathology.

However, it is worth noting that there were no significant differences in the diagnostic sensitivity of EUS-FNA puncture pathology when subgroups were analyzed based on factors such as the presence of comorbidities (eg, acute pancreatitis, acute cholangitis, choledochal stones), the thickness of the puncture needles, the manufacturer of the operative equipment, the duration of the EUS-FNA operation, and the proficiency of the operator [Table 4]. In addition, refer to Supplemental Table 1, http://links.lww.com/ENUS/A363, for the basic information, laboratory test results, imaging data, and surgical details of patients using different EUS-FNA puncture needles.

### IgG4-related disease

Among our cohort of 118 patients diagnosed with benign DBSs, a notable subset of 33 individuals (28%) received a diagnosis of IgG4-RD. Of these cases, 26 were male patients, whereas the remaining 7 were female patients, with male patients averaging 60.73 years in age and female patients averaging 64.46 years [Table 1]. In terms of disease manifestation, we observed that 20 cases exhibited involvement in a single region, including the head and neck, chest, hepatobiliary pancreas, or retroperitoneum. Thirteen cases presented with multiple region involvement, and intriguingly, 9 male patients displayed involvement across multiple regions.

Serum IgG4 levels in patients diagnosed with IgG4-RD exhibited a mean value of 8.69 g/L. Notably, IgG4 levels fell within the normal range for 4 cases, were 1 to 2 times the upper limit in 8 patients and 2 to 5 times the upper limit in 12 patients, and exceeded 5 times the upper limit in 9 cases. In comparison with other etiologies of DBSs, it is significant to highlight that patients with IgG4-RD presented elevated transaminase and bilirubin levels (*P* < 0.05). Moreover, tumor marker levels demonstrated a notable elevation in IgG4-RDs, with a median CA-199 level of 69.30 U/L. [Table 1].

**Figure 2:**
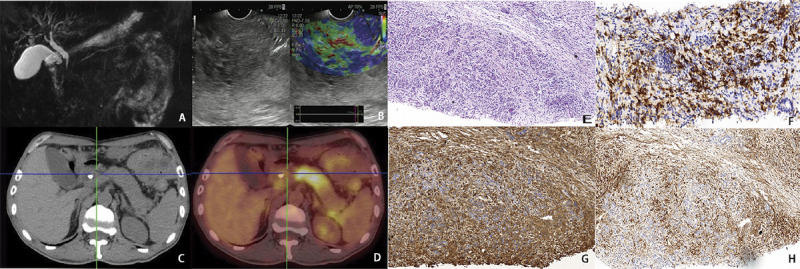
Imaging manifestations and pathologic manifestations of IgG4-related diseases. A, MRCP: the lumen of the pancreatic segment of the common bile duct appears narrowed, forming a thin line. This is accompanied by dilatation of the bile ducts both above and below it, including the intrahepatic and extrahepatic bile ducts. The widest part of the common bile duct measures approximately 1.7 cm. B, EUS: in the head of the pancreas, there is a hypoechoic area with irregular but well-defined borders. Its cross-sectional size measures approximately 3.98 × 1.96 cm. This area exhibits medium elasticity and a characteristic imaging texture. C, CT: the pancreas displays diffuse swelling, along with thickening of the walls in the middle and upper bile ducts. D, PET/CT: the bile duct wall shows increased FDG uptake with an SUV_max_ of 2.4. Nodular increased FDG uptake is particularly noticeable in the neck and body of the pancreas on conventional scans, with an SUV_max_ of 5.0. FDG uptake remains persistently increased on delayed scans, reaching an SUV_max_ of 6.0. E, Pancreatic EUS-FNA puncture (HE stain): the findings indicate diffuse chronic inflammatory cell infiltration, along with the proliferation of interstitial fibrous tissue and sclerosis. Pancreatic ductal hyperplasia is also observed. F, Pancreatic EUS-FNA puncture (CD138): scattered positive results are observed. G and H, Pancreatic EUS-FNA puncture (IgG and IgG4): in some areas, the IgG4/IgG ratio exceeds 40%, and the IgG4 count is greater than 10 HPF. CT: computed tomography; FDG: fluorodeoxyglucose; HE: hematoxylin and eosin; HPF: high-power field; MRCP: magnetic resonance cholangiopancreatography; PET/CT: positron emission tomography/computed tomography; SUV_max_: standardized uptake value.

Radiological imaging of the biliopancreatic system among patients with IgG4-RDs in our study consistently revealed diffuse pancreatic swelling, suspicious thickening of the wall of the pancreatic segment of the common bile duct, luminal stenosis, dilatation of the upstream bile ducts, and diffuse swelling in other regions. Positron emission tomography/CT scans further suggested nodular increases in FDG metabolism [Figure 2].

It is noteworthy that histopathologic specimens of the pancreaticobiliary duct obtained by EUS-FNA puncture in only 9 patients showed histopathologic manifestations typical or partially typical of IgG4-RD (dense lymphocytic infiltrate and storiform fibrosis with or without obliterative phlebitis; the IgG4^+^:IgG^+^ ratio is ≥41%, and/or the number of IgG4^+^ cells/high-power field is ≥10) [Figure 2].

## DISCUSSION

Indeterminate DBSs present a diagnostic challenge. We examined EUS-FNA cytology and histology for suspected malignant DBSs in this study. The sensitivity of EUS-FNA cytology, histology, and codiagnosis was 69.1%, 76.4%, and 82%, respectively, and the diagnostic sensitivity was 97.5%, 99.1%, and 99.1%, respectively. The diagnostic efficacy, in terms of both sensitivity and specificity, remained consistent for EUS-FNA cytology and histology, irrespective of considering instances of histological slide failure. Furthermore, the origin of DBSs—whether stemming from bile duct, pancreatic, or duodenal papillary lesions—did not significantly impact the diagnostic efficacy of EUS-FNA. Interestingly, we observed heightened diagnostic efficacy associated with EUS-FNA in cases where a discernible mass shadow surrounded the DBSs, particularly when this shadow exhibited larger dimensions. Additionally, benign DBSs due to IgG4-RDs accounted for 28% of cases, a crucial factor in the differential diagnosis.

Numerous studies have addressed DBSs, yet varying anatomical definitions persist. Commonly used delineations encompass the distal third of the extrahepatic bile duct, the intrapancreatic segment of the common bile duct, or the distal half of the extrahepatic bile duct.^[24–26]^ In this article, we adopt a precise definition for distal bile duct stricture: the bile ducts distal to the posterior duodenum and proximal to the confluence of the cystic duct with the common bile duct. This definition aligns with that of distal CCA in the joint manual of cancer staging issued by the American Joint Committee on Cancer and the Union for International Cancer Control.^[1]^ Its anatomical clarity simplifies clinical application.

For DBSs, selecting the appropriate tissue acquisition method is crucial. ERCP-guided tissue acquisition is recommended when biliary drainage is necessary.^[24]^ However, when cross-sectional imaging indicates a mass associated with the stricture or when ERCP-guided tissue acquisition fails, EUS-FNA becomes the preferred option.^[24]^ In our study, about 20% of patients required ERCP for biliary stent placement or endoscopic nasobiliary drainage for drainage. Sensitivities for ERCP-guided brushings, biopsies, and combined brushings and biopsies in this group were 50%, 70%, and 77.8%, respectively. These results align with the diagnostic sensitivity range (43%–81%) reported in existing literature for ERCP.^[9–12]^ In contrast, EUS-FNA offers distinct advantages, particularly in diagnosing mass-type lesions, which are prevalent in DBSs with a primary pancreatic etiology. Among 146 patients with imaging suggestive of a mass shadow, EUS-FNA cytology, histology, and combined cytology and histology demonstrated diagnostic sensitivities of 75.9%, 79.2%, and 85.8%, respectively—outperforming cases with solely bile duct stricture on imaging. This finding corroborates previous studies. In a meta-analysis of 4 studies encompassing 158 patients, EUS-FNA exhibited a pooled sensitivity and specificity of 83% (95% CI, 68%–98%) and 100% (95% CI, 63%–100%) for DBSs, comparable to our study’s sensitivity (82%) and specificity (99.1%) for EUS-FNA cytology combined with histology in DBS diagnosis.^[27]^ Notably, EUS-FNA’s diagnostic performance excels in identifying pancreatic masses, with pooled sensitivity and specificity of 86.8% and 95.8% in a meta-analysis.^[28]^ In cases of biliary stricture, EUS-FNA yields a sensitivity of 80% and specificity of 97% for characterizing the stricture.^[27]^ These findings underscore EUS-FNA’s utility in DBS diagnosis, particularly when mass lesions are involved, aligning with prior research.

During EUS-FNA procedures, practitioners are particularly concerned with the choice of needle type. In DBSs, the 2 most frequently used needle types are 22-gauge and 25-gauge. Tomoda and colleagues’^[29]^ study found that for 88 patients with pancreatic lesions, the 25-gauge Franseen needle was as effective as the 22-gauge Franseen needle in tissue acquisition and showed no significant difference in diagnostic performance. This finding aligns with the results of Gimeno-García and colleagues’ study on 120 cases of gastrointestinal and adjacent organ solid lesions.^[21]^ In terms of diagnostic sensitivity and specificity, our study’s outcomes were consistent with previous research. However, we did not evaluate the quality of the obtained specimens. Consequently, based on these research findings, the 25-gauge needle seems to have a comparative advantage in the selection of needle type.

EUS-FNA is a multistep procedure influenced by various uncertain factors, resulting in variability in pathology sampling quality.^[30–32]^ Our study alone observed suboptimal sampling in 6.8% of patients, highlighting a need for improved techniques. One potential solution is ROSE, involving real-time assessment of sample adequacy and diagnostic yield.^[33]^ However, the debate surrounding the necessity of ROSE persisted for 2 decades. A meta-analysis of 34 studies aimed to determine whether ROSE impacts the diagnostic accuracy of EUS-FNA in solid pancreatic lesions.^[34]^ The findings revealed that ROSE remained an independent determinant of EUS-FNA accuracy (*P* = 0.001). Yet, a systematic review by Kong et al.,^[35]^ encompassing 7 studies with 1299 patients, indicated that EUS-FNA with ROSE did not enhance diagnostic yield or adequacy. Furthermore, attempts to reduce ROSE’s labor costs led some centers to explore whether endoscopists could replace cytologists for evaluation. However, results have been unfavorable. A double-blind prospective controlled trial demonstrated that even well-trained endoscopists were less accurate than cytopathologists in assessing specimen adequacy (*P* = 0.004) and providing a preliminary estimate of malignancy (*P* < 0.001).^[36]^ Looking ahead, advancements in deep-learning techniques and increased computational power hold promise for the application of artificial intelligence in enhancing ROSE accuracy while reducing costs.^[37]^ This avenue may lead to more precise and cost-effective ROSE procedures in the future.

EUS plays a pivotal role not only in obtaining pathological tissue for diagnostic purposes but also in using advanced techniques such as EUS elastography (EUS-E) and contrast-enhanced EUS (CE-EUS) to broaden its diagnostic capabilities in the context of DBSs. Both EUS-E and CE-EUS offer qualitative, semiquantitative, and quantitative methods for the differential diagnosis of DBSs.^[38]^ Although EUS-E and CE-EUS have been extensively studied in the context of pancreatic lesions, with several meta-analyses demonstrating high sensitivity (92%–98%), their specificity in diagnosing malignant pancreatic tumors remains relatively low (67%–76%).^[39]^ Notably, Kitano et al.^[40]^ focused on CE-EUS and reported pooled estimates of sensitivity and specificity at 93% (91%–95%) and 80% (75%–85%), respectively, for this clinical application. It is worth highlighting that there is a dearth of reports on the use of EUS-E and CE-EUS specifically for the assessment of bile duct occupancy alone within the existing literature. This underscores the potential for further exploration and research in this important diagnostic area.

It is important to recognize that the growing awareness of IgG4-RD and the establishment of globally recognized diagnostic criteria have led to an increasing prevalence of bile duct stenosis attributed to IgG4-related sclerosing cholangitis.^[41]^ This entity now represents a significant portion of benign bile duct stenosis cases and warrants careful consideration in the differential diagnosis. In our study, we diagnosed 33 cases of bile duct stenosis attributed to IgG4-RDs, comprising 28% of all benign bile duct stenosis cases. Diagnosing IgG4-related sclerosing cholangitis poses a clinical challenge, as it can manifest with markedly elevated tumor marker levels and limited stenosis on imaging when other extrabiliary organs remain unaffected.^[42]^ This can easily lead to misdiagnosis as a malignant stenosis. A comprehensive diagnostic approach should encompass typical histopathologic, clinical, serologic, and imaging findings when assessing IgG4-RD. Diagnostic criteria for this condition have evolved from organ-specific and general benchmarks in the 20th century to the 2019 ACR/EULAR classification criteria.^[43,44]^ In a multicenter retrospective study of patients with kidney disease, these latest ACR/EULAR criteria demonstrated strong performance (sensitivity 90.9%, specificity 98%, PPV 98%, and NPV 90.7%).^[45]^ However, further prospective studies are necessary to validate these criteria in clinical practice.

Our study had several limitations. First, the study did not uniformly use ROSE during EUS-FNA procedures. ROSE can enhance the assessment of sample adequacy and diagnostic yield. However, its inconsistent use in this study may have impacted the diagnostic accuracy and completeness of results. Second, this study was conducted in a single center, which might introduce selection bias and limit the generalizability of findings. Multicenter studies with diverse patient populations could provide a broader perspective on the diagnostic performance of EUS-FNA. Moreover, although this study identified cases of bile duct stenosis due to IgG4-RDs, diagnosing these conditions can be challenging due to their varied clinical presentations and imaging findings. The criteria used were based on the 2019 ACR/EULAR classification criteria, but further prospective validation studies are needed to confirm their utility. These limitations should be considered when interpreting the results and applying them to clinical practice.

In summary, our investigation substantiates the pivotal role of EUS-FNA within the diagnostic framework for DBSs. The robust diagnostic sensitivity and specificity exhibited by EUS-FNA reinforce its significance as an invaluable diagnostic modality in this domain. Our analysis identified that the primary determinants influencing the diagnostic efficacy of EUS-FNA were the presence or absence of a discernible mass shadow surrounding the DBSs and the dimensions of this observed mass shadow. Furthermore, our findings emphasize the relevance of considering IgG4-RDs, which constitute a substantial portion of cases, during the differential diagnosis of these strictures.
